# One-quarter of individuals with weekly headache have never consulted a medical doctor: a Danish nationwide cross-sectional survey

**DOI:** 10.1186/s10194-022-01460-6

**Published:** 2022-07-18

**Authors:** Thien Phu Do, Simon Stefansen, Mikala Dømgaard, Timothy J. Steiner, Messoud Ashina

**Affiliations:** 1 Danish Headache Center, Department of Neurology, Rigshospitalet Glostrup, Faculty of Health and Medical Sciences, University of Copenhagen, Copenhagen, Denmark; 2 Danish Knowledge Center On Headache Disorders, Rigshospitalet Glostrup, Valdemar Hansens Vej 2, 2600 Glostrup, Denmark; 3 Department of Neuromedicine and Movement Science, Norwegian University of Science and Technology, Trondheim, Norway; 4 Division of Brain Sciences, Imperial College London, London, UK

**Keywords:** Headache disorders, Disease burden, Healthcare utilization, Barriers to care, Population survey, Denmark

## Abstract

**Introduction:**

Large numbers of people with headache who would benefit are not reached by headache services. Among the causes are poor or disorganized provision of headache services, but reluctance to seek healthcare has frequently been identified as a significant barrier. We conducted a national survey of people with headache to assess the extent of this problem in Denmark, a country with well organized, highly resourced, and readily accessible services.

**Methods:**

We conducted a nationwide cross-sectional survey of adults ≥ 18 years old in Denmark reporting at least one headache day in the last year. We used social media (Facebook) to publicize and drive a recruitment campaign. The survey investigated five items: (1) disease burden, (2) social life, (3) presenteeism, (4) social support, and (5) healthcare utilization.

**Results:**

We included 6,567 respondents from May 2021 to June 2021; 70.2% were female, 39.8% male, and mean age was 43.2 ± 13.4 years. Of the respondents, 54.2% reported headache at least once a week, 33.4% reported headache a couple of times a month, and 12.4% reported headache a couple of times a year. Two-thirds of respondents (66.6%) reported that headache limited their social lives occasionally or frequently. Most respondents (86.8%) reported going to work or attending educational activities occasionally or more frequently even though they had headache. Half of the respondents (49.5%) experienced lack of understanding of their headaches from people occasionally or more frequently. Almost half of respondents (43.7%) had never consulted a medical doctor for their headache; even of those with weekly headache, more than a quarter (28.3%) had never done so in their lifetimes.

**Conclusions:**

Headache disorders continue to be a problem, even in a high-income country with free and easily accessible headache services. Further studies are needed to investigate and clarify why even people with the highest burden are hesitant to seek and make use of widely available headache services.

## Key messages

- In Denmark it is evident that many people, even among those with the highest headache-attributed burden, are hesitant to make use of headache services that offer mitigation of symptoms and their consequences of disability, lost productivity, and economic losses.
- It is insufficient merely to make headache services available: public education – in when, how and when not to use these services – is also needed if they are to reach all (or even the majority) of those who would benefit.

## Introduction

Headache disorders are a leading cause of disability, directly affecting more than 1 billion people across all regions of the world [1]. In Denmark, headache disorders are responsible for more than one-third of all disability-adjusted life years (DALYs) due to neurological disorders according to the *Global Burden of Disease study* [2]. Because the most common headache disorders, tension-type headache and migraine, are highly prevalent during the most productive years of life [1, 3, 4], the impacts and importance of this disability burden are magnified. These are extensive and include lifestyle compromises and impaired interpersonal relationships [1, 3]. Despite this, and the existence of effective treatments, multiple studies highlight failure of healthcare to deliver adequate care [1, 3]. Large numbers of people with headache who would benefit are not reached by headache services. Among the causes are poor or disorganized provision of headache services, but reluctance to seek healthcare has frequently been identified as a significant barrier [1, 3, 5, 6]. We conducted a national survey of people with headache to assess the extent of this problem in Denmark, a country with well organized, highly resourced, and readily accessible services.

## Methods

This was a national cross-sectional survey. Data were collected during May and June 2021 using the software SurveyXact (Rambøll Group A/S, Copenhagen).

### Ethics

Health surveys in Denmark do not require approval by the National Committee on Health Research Ethics (or any other ethics committee). Consent of participants was presumed from their participation following explanation of the nature and purpose of the survey (the latter broadly expressed as an assessment of life with headache and its impacts). Data were handled confidentially and in accordance with data protection legislation, with anonymity of respondents preserved throughout.

### Screening and recruitment

We used social media (Facebook) to publicize and drive a recruitment campaign, with no predetermined prioritization of age or gender. Users of Facebook in Denmark aged ≥ 18 years were therefore the sampled population. Of 4.8 million people in Denmark meeting the age criterion, approximately 3.3 million (69%) were estimated to have a Facebook account [7].

Eligible participants were those meeting the age criterion and reporting at least one headache day in the last year. Respondents received no remuneration for participation.

### Assessments

The questionnaire was developed by the Danish Knowledge Center on Headache Disorders, a public institution focused on raising levels of awareness and knowledge about headache disorders among healthcare providers and people affected by them. The survey consisted of the following five items (Table 1):

1. **Disease burden:** How often do you have headache? (Headache frequency was assessed through a 1-year recall of headache days. Participants were asked to choose one of three categories according to best match: at least once a week, a couple of times a month or a couple of times a year.)
2. **Social life:** Does your headache disorder limit your social life? (Never, rarely, occasionally, often, very often)
3. **Presenteeism:** Do you ever go to work/attend educational activities even though you have a headache? (Not relevant, never, rarely, occasionally, often, very often)
4. **Social support:** Do you experience a lack of understanding of your headache from people around you? (Never, rarely, occasionally, often, very often)
5. **Healthcare utilization:** Have you consulted a medical doctor about your headache? (Yes, no)

**Table 1:** Responses to the enquiry

Enquiry	Response according to reported headache frequency			
**How often do you have headache?**	**All respondents** *N* = 6,567 (100%)	**At least once a week** *n* = 3,558 (54.2%)	**A couple of times a month** *n* = 2,195 (33.4%)	**A couple of times a year** *n* = 814 (12.4%)
**Age, years**				
Mean ± SD	43.2** ± **13.4	41.2** ± **13.1	42.8** ± **12.7	48.9** ± **14.9
	**Proportion, n (%)**			
**Gender**				
Male	1,956 (29.8%)	936 (26.3%)	603 (27.5%)	417 (51.2%)
Female	4,611 (70.2%)	2,622 (73.7%)	1,592 (72.5%)	397 (48.8%)
**Does your headache limit your social life?**				
Never	1,750 (26.6%)	169 (4.7%)	222 (10.1%)	440 (54.1%)
Rarely	1,571 (23.9%)	512 (14.4%)	610 (27.8%)	237 (29.2%)
Occasionally	3,009 (45.8%)	1.805 (50.7%)	1,101 (50.2%)	103 (12.7%)
Often	967 (14.7%)	741 (20.8%)	206 (9.4%)	20 (2.5%)
Very often	400 (6.1%)	331 (9.3%)	56 (2.6%)	13 (1.6%)
**Do you ever go to work or attend educational activities even though you have a headache?**				
Never	127 (1.9%)	45 (1.3%)	23 (1.0%)	59 (7.2%)
Rarely	188 (2.9%)	30 (0.8%)	58 (2.6%)	100 (12.3%)
Occasionally	793 (12.1%)	219 (6.2%)	396 (18.0%)	178 (21.9%)
Often	1,446 (22.0%)	779 (21.9%)	582 (26.5%)	85 (10.4%)
Very often	3,463 (52.7%)	2,228 (62.6%)	1,040 (47.4%)	195 (24.0%)
Not relevant	550 (8.4%)	257 (7.2%)	96 (4.4%)	197 (24.2%)
**Do you experience a lack of understanding of your headache from people around you?**				
Never	1,750 (26.6%)	510 (14–3%)	656 (29.9%)	584 (71.7%)
Rarely	1,571 (23.9%)	784 (22.0%)	652 (29.7%)	135 (16.6%)
Occasionally	2,140 (32.6%)	1,411 (39.7%)	663 (30.2%)	66 (8.1%)
Often	787 (12.0%)	595 (16.7%)	170 (7.7%)	22 (2.7%)
Very often	319 (4.9%)	258 (7.3%)	54 (2.5%)	7 (0.9%)
**Have you consulted a medical doctor about your headache?**				
Yes	3,695 (56.3%)	2,550 (71.7%)	1,025 (46.7%)	120 (14.7%)
No	2,872 (43.7%)	1,008 (28.3%)	1,170 (53.3%)	694 (85.3%)

We excluded participants who did not complete all questions.

### Statistical methods

We used descriptive statistics to present survey demographics and headache-attributed burden, calculating means and standard deviations (SDs) for continuous outcomes and proportions (%) for binary and multinomial outcomes. Descriptive analyses were conducted using Microsoft Excel, version 2103 (16.0.13901.20400) / April 13, 2021.

The strength of the association between headache-attributed burden (at least once a week, a couple of times a month, a couple of times a year) and survey outcomes of ‘Does your headache limit your social life?’, ‘Do you ever go to work or attend educational activities even though you have a headache?’, or ‘Do you experience a lack of understanding of your headache from people around you?’ were assessed with the Gamma (G) coefficient (Goodman and Kruskal's gamma); A G value of 1.00 represents a perfect positive association, a value of 0.00 reflects no association, and a value of -1.00 reflects a perfect negative association between the variables. For the question ‘Do you ever go to work or attend educational activities even though you have a headache?’, entries with ‘Not relevant’ were excluded from the analysis. The G coefficient was calculated using IBM SPSS Statistics, version 28.0.0.0 (190).

## Results

### Study population

**Fig. 1:**
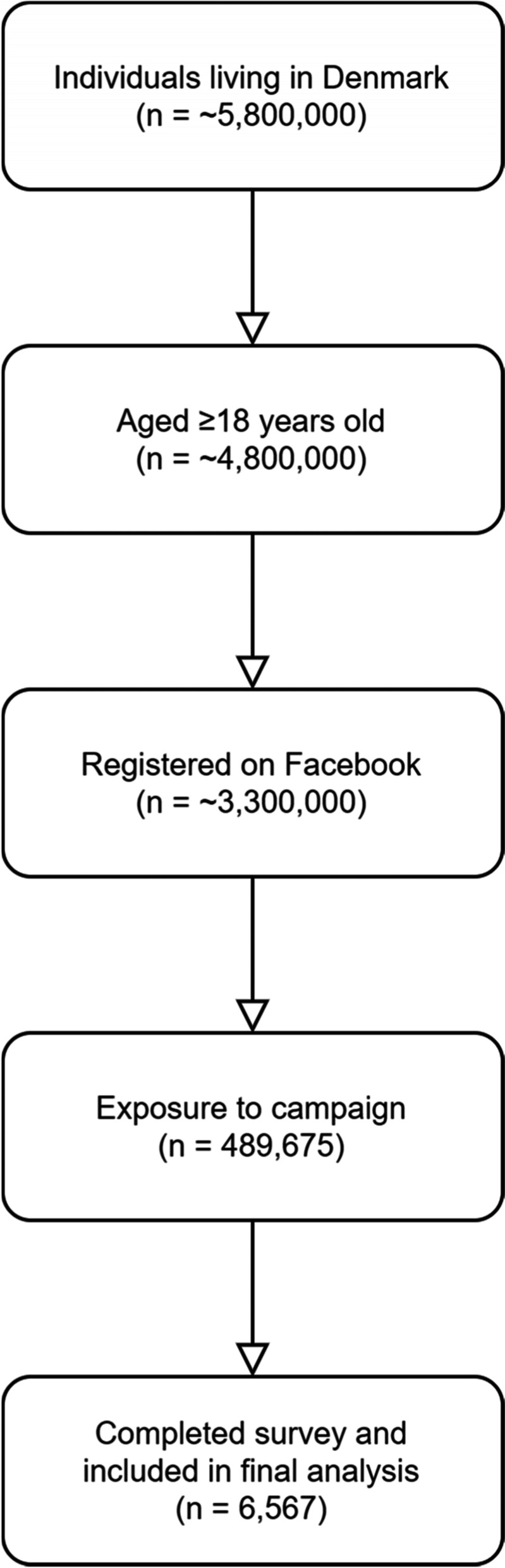
Participant flow diagram

The screening and recruitment campaign had more than 2.1 million exposures, reaching 489,675 unique users. Of these, 6,567 (1.34%) completed all questions and were included in the analysis (Fig. 1). However, the number who engaged with the survey, making a decision to respond or not, could not be determined. Consequently, as in internet surveys generally, the denominator and participating proportion were unknown.

### Respondent demographic

**Fig. 2:**
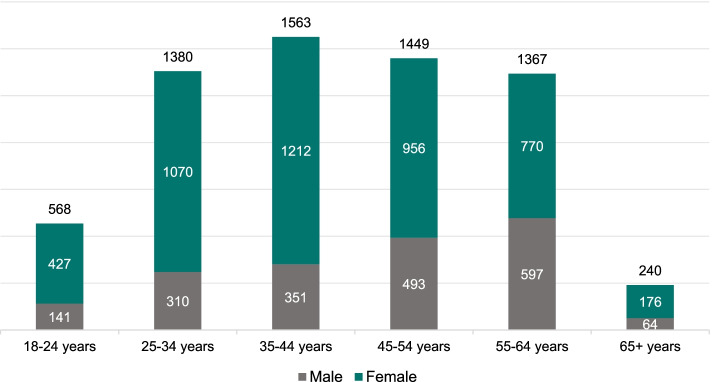
Age and gender distribution of participants (n)

Of the 6,567 respondents, 4,611 (70.2%) were female and 1,956 (39.8%) male, with a mean age of 43.2 ± 13.4 years (Fig. 2).

### Headache-attributed burden

Of the 6,567 respondents, 3,558 (54.2%) reported headache at least once a week, 2,195 (33.4%) reported headache a couple of times a month, and 814 (12.4%) reported headache a couple of times a year.

### Impact on social life

Two thirds of respondents (66.6%) reported that headache limited their social life occasionally or frequently (Table 1). Of those with headache at least once a week, most (80.8%) reported occasional or more frequent social-life limitations as a consequence. Those with less frequent headache were less likely to do so: two thirds (62.2%) of respondents with headache monthly and much fewer (16.8%) of those with headache only a couple of times a year. There was a strong positive correlation between headache-attributed burden and limitation of social life (G = 0.576, *p* < 0.0001).

### Presenteeism

There was a high rate of presenteeism. Most respondents (86.8%) reported going to work or attending educational activities occasionally or more frequently even though they had headache (Table 1). Again, this was frequency-dependent: of respondents with weekly or monthly headache, almost all (90.7% and 91.0%, respectively) reported doing so, but fewer, although still more than half (56.3%) of those with yearly headache did so. There was a strong positive correlation between headache-attributed burden and presenteeism (G = 0.519, *p* < 0.0001).

### Social support

Half of the respondents (49.5%) experienced lack of understanding of their headaches from people around them occasionally or more frequently (Table 1): almost two thirds (63.7%) of those with weekly headache, two fifths (40.4%) of those with monthly headache and fewer (11.7%) of those with yearly headache. There was a strong positive correlation between headache-attributed burden and lack of social support (G = 0.448, *p* < 0.0001).

### Healthcare utilization

Almost half of respondents (43.7%) had never consulted a medical doctor for their headache (Table 1). Even of those with weekly headache, more than a quarter (28.3%) had never done so, while over half with monthly headache (53.3%) and a large majority with yearly headache (85.3%) also had never done so.

## Discussion

In this national survey of people with headache in Denmark, a high-income country with readily accessible services, we identified a low healthcare utilization rate. Even among those with the highest disease burden (weekly headache), one-quarter had never consulted a medical doctor in their lifetimes. This was despite a sampling method that, very probably, introduced a strong selection bias towards those most concerned about headache, and, presumably, most likely to consult. Utilization rate among the general Danish population with headache is almost certainly lower – and perhaps much lower – than in our sample. Furthermore, headache-attributed burden was strongly associated with a negative impact on social life, higher rates of presenteeism, and lack of understanding from those around them.

In a web-based survey of panelists with migraine from six different countries (United States, Canada, France, United Kingdom, Germany, and Australia), only about one-third of those with chronic migraine in the United States (who have the greatest disease burden) had visited a healthcare provider for their headache within the past three months [5]. Among those with episodic migraine, the healthcare utilization rate was even lower (one-fifth of US-based individuals with episodic migraine) [5]. While other countries in the study reported higher rates, there were still substantial proportions of people with headache who did not seek headache care [5, 6]. In general, as we found, the lower the disease burden, the lower the probability of consultation, but even high burden does not raise it to anywhere near 100%.

The underlying reasons are certainly multifactorial. Clinical, social, and political/economical barriers hinder people with headache from accessing healthcare services who would otherwise benefit from them [1, 8]. Inadequate training of healthcare providers constitutes the principal clinical barrier, with limited training in headache medicine during medical school that continues into residency [8, 9]. Reports from other countries consistently demonstrate that there is room for improvement of services, in which diagnostic delay, misdiagnosis, unsatisfactory management and suboptimal outcomes continue to affect headache care [1]. In one transcontinental study of people with migraine who had been referred to tertiary care, the initial diagnosis was incorrect in more than two-thirds [10], inevitably undermining subsequent management. Delays and poor outcomes are highly discouraging, resulting in reduced expectations and diminished enthusiasm for utilizing headache services. Danish headache services are considered to be among the best in Europe [11]¸ but are unlikely to be entirely free of these factors (data specific for Denmark are unavailable).

Social barriers include poor awareness of headache and a tendency among the general public to perceive headache disorders as not serious. They do not cause death, but this perception has much deeper roots than this: many people do not give credence to the possibility that headache can be significantly disabling. Both structural and functional qualities of a social network are associated with health [12, 13]. A social network exerts pressure on each member to adapt their health behavior to the consensus of the group [14]. But stigmatization and trivialization of headache as a minor annoyance confront people with headache [1, 15, 16]. An inevitable consequence of negative attitudes towards headache is a to higher rate of presenteeism (remaining work despite having headache) [17, 18]. In support of this proposition, half of our respondents reported some level of lack of understanding – real or perceived – from those around them. They act as deterrents, even in wealthy nations and even among those with high disease burden, from seeking headache care [1]. Very probably, they also contribute to a high rate of presenteeism.

Political barriers include governmental failure to give due priority to headache services, and to allocate the resources they require [1]. Economic barriers operate at both provider and consumer levels. Governments are dissuaded by the cost of universal headache care, overlooking the ill-health and lost-productivity penalties of inaction which would be greatly offset by better care [8]. While the economic benefits of good headache care accrue also at individual level, and are potentially cost saving even for those with limited financial resources [19], the need to pay for care can be challenging. Lack of universal healthcare coverage, or insurance, is a barrier to care in the United States, for example, while the potential loss of pay may contribute to high rates of presenteeism there [5]. But Denmark provides free access to healthcare, and, in theory, universal coverage for all its residents, so the explanations of our findings have to be found elsewhere.

### Strengths and limitations

A strength of the study was the relatively large sample size of > 6,500 respondents in a country with a population of fewer than 5 million adults. Another was the anonymity of the respondents, which allowed us to request information that people might not otherwise feel comfortable discussing. Both of these derived from the sampling method. However, this also conferred limitations. We could not relate our findings to detailed demographics characteristics of respondents, or they medical histories, since we only enquired only into age and gender. The likely biases imposed by the sampling method and participating proportion have been identified already. However, these almost certainly led to conservative findings, at least regarding failure to consult, which in all probability was under-estimated. While a large population of Denmark are Facebook users, not all of the population will be equally represented among these: in particular, older adults (above middle age) are less likely to have been exposed to the recruitment campaign [7]. These are people with a lower probability of having headache, or, if they do, of having a high attributable burden. In addition, categorizing disease burden, as we did for simplification (at least once a week, a couple of times a month or a couple of times a year), might have been a limiting factor in estimation of disease burden.

## Conclusions

In Denmark it is evident that many people, even among those with the highest headache-attributed burden, are hesitant to make use of headache services that offer mitigation of symptoms and their consequences of disability, lost productivity, and economic losses. Lack, or poor quality, of headache services are undoubtedly factors elsewhere in failure to seek headache care. However, in Denmark, a high-income country with headache services that are free, easily accessible and among the best in the world, the main drivers must be found elsewhere. If clinical and political/economical barriers are not to blame, it is tempting to attribute the cause to social barriers, but it would be simplistic to draw such a conclusion from this study and the limited other evidence available. Qualitative studies, engaging directly with those affected, are needed to understand the causes. What is clear meanwhile is that it is insufficient merely to make headache services available: public education – in when, how and when not to use these services – is also needed if they are to reach all (or even the majority) of those who would benefit [20].

## Abbreviations

DALY: Disability-adjusted life year
SD: Standard deviation
